# Lady Dufferin's Fund

**Published:** 1890-02-15

**Authors:** 


### Lady Dufferin's Fund.

The fifth annual general meeting of Lady Dufferin's Fund was held at the Calcutta Town Hall on Friday, the 7th inst., the Viceroy presiding. There was a large attendance, including many European ladies and gentlemen and several members of the Hindoo and Mahomedan aristocracy. The Honourable Mr. Hutchins presented the report of the Central Committee, which records a steady and satisfactory expansion of the work of the association during the first year of Lady Lansdowne's tenure of office as lady president. The formation of a branch in the United Kingdom is described as one of the most interesting and important events of the year, while the decoration of Lady Dufferin with the Order of Victoria and Albert is a further sign, if any be wanting, of the warm interest taken by her Majesty in the work. The organisation of a provincial branch in Beloochistan is also mentioned. A number of small local branches have also been formed in various towns throughout the country. A list is given of 238 students who are studying at different medical schools, and it is mentioned that several past students are now doing well in private practice or in connexion with institutions under the fund. It is added that many native States are sending students to be trained at the central colleges, and several acts of liberality in the foundation of scholarships and the giving of prizes are also mentioned.

Mr. Hutchins spoke at some length. He described the progress of the Association throughout the country, made an earnest appeal for the half lakh of rupees still needed for the buildings of the Dufferin Zenana Hospital, and concluded by thanking the lady president, who, with the aid of the honorary secretaries, had borne the brunt of the daily work and correspondence.

Justice Ameer Ali, in moving the adoption of the report, expressed regret that Bengal, and especially Calcutta, had not hitherto given the movement the same enthusiastic support as other parts of the country. The tree planted by a large-hearted English lady, he added, which, under the care of another, was now spreading its branches all over the land, needed but native help to take root firmly in the soil.

The Honourable Rash Behary Ghose, in seconding the motion, said India was distinguished for its charity, and the only thing now needed was its diversion into new channels.

The Honourable Mr. Nulkar followed with a long speech, in the course of which he expressed confidence that the day would soon come when the women of India would wonder how they ever got on without professional help from their own sex.

Mr. Anundo Mohun Bose said if the voice of India could be heard that evening, then in every accent and every tongue spoken would rise a chorus of gratitude for the services of the association.

The Lieutenant-Governor gave a short account of the work of the Bengal branch, and mentioned several instances of individual liberality, such as that of the Maharajah of Durbhungah, who had established a complete hospital at his own headquarters, and that Rai Dhunput Singh Bahadur had offered to bear the entire cost of a female dispensary at Moorshedabad. With such instances of liberality, was it impossible to raise a lakh of rupees in Calcutta to finish the hospital which bore the honoured name of the foundress of the association? He concluded by moving a vote of thanks to the Viceroy for presiding.

The motion was seconded by the Marharajah of Durbhungah.

The Viceroy, after assuring the meeting of the deep interest he took in the welfare of the association, said the past year was one of special interest for Lady Landsdowne and himself, as the first year she had occupied the position of lady president. He thought the association had now passed out of the period of emotion, sentiment, and excitement, and had steady-going business-like surroundings. He confidently hoped that it had gone safely through all the ailments of infancy, and was now entering what promised to be a robust and vigorous youth. After referring to some of the most gratifying features of the report, he proceeded to express Lady Lansdowne's deep gratitude to the ladies and gentlemen who had so loyally assisted her, and her deep regret that the association was about to lose in Lady Reay one of its most energetic and intelligent supporters.

